# Reasons for Indoor and Outdoor Tanning: Starting Points for Skin Cancer Prevention Based on a Nationwide Study

**DOI:** 10.3390/curroncol33050257

**Published:** 2026-04-29

**Authors:** Katharina Diehl, Lisa Voß, Eckhard W. Breitbart, Inga-Marie Hübner, Tatiana Görig

**Affiliations:** 1 Department of Medical Informatics, Biometry and Epidemiology, Friedrich-Alexander-Universität Erlangen-Nürnberg (FAU), 91054 Erlangen, Germany; 2 Bavarian Cancer Research Center (BZKF), 91054 Erlangen, Germany; 3 Comprehensive Cancer Center Erlangen-European Metropolitan Area of Nürnberg (CCC ER-EMN), 91054 Erlangen, Germany; 4 Association of Dermatological Prevention (ADP), 21614 Buxtehude, Germany

**Keywords:** UV exposure, tanning, sunbed, tanning bed, motivation, reasons, outdoor, skin cancer, risk behavior, ultraviolet radiation

## Abstract

Background: Ultraviolet (UV) radiation is a major risk factor for skin cancer. Nevertheless, many people tan in the sun and in tanning beds. The aim of this study was to explore the reasons behind these behaviors and to investigate whether the reasons for tanning in the sun and in tanning beds differ. Methods: We used data from a nationwide survey study conducted in Germany, which included n = 4156 individuals aged 16 to 65 years. We assessed different reasons for outdoor and indoor tanning, as well as sociodemographic characteristics, skin type, and tanning behaviors. Results: While both outdoor and indoor tanners frequently reported relaxation, as well as feeling of light and warmth as reasons, outdoor tanners placed a greater emphasis on increasing vitamin D levels and health benefits. In contrast, indoor tanners were more focused on enhancing attractiveness and pre-tanning for holidays. Individuals who sought a tan more frequently—whether through indoor or outdoor tanning—were more likely to agree with the various reasons provided, compared to those who tanned less often. In addition, we found associations with sex, age, immigrant background, education, occupation, and skin type. Conclusions: There were differences as well as similarities in the reasons for indoor and outdoor tanning. This indicates that overarching prevention strategies could be effective. Additionally, targeted measures specifically for indoor and outdoor tanning could also be beneficial in raising awareness about the risks of UV radiation and, in the long term, reducing the incidence of skin cancer.

## Simple Summary

Ultraviolet radiation, also known as UV radiation, is a risk factor for the development of skin cancer. This applies to both the natural UV radiation from the sun and the artificial UV radiation from tanning beds. We examined the reasons for tanning in the sun and in tanning beds to better understand the motivation behind it. The results can help develop targeted prevention measures to raise awareness about the potential risks of UV radiation. In the long term, these measures could help reduce the number of new skin cancer cases.

## 1. Introduction

The incidence of skin cancer is rising in Western countries [1,2,3]. Ultraviolet (UV) radiation is a major risk factor for the development of skin cancer [4,5,6,7,8]. Despite this, many individuals in Western countries intentionally expose themselves to UV radiation through outdoor sunbathing and indoor tanning. UV radiation from the sun and tanning beds has been classified as a Group 1 carcinogen for humans [4]. As a result, intentional tanning represents a largely overlooked public health challenge, as it constitutes a preventable risk factor for skin cancer [9,10]. Thus, an important question arises: why do people intentionally seek to tan?

The current state of research suggests that there may be differences in the motivations for indoor versus outdoor tanning. Regarding indoor tanning, most frequently named reasons were enhancing attractiveness, including sex appeal, feeling to have a skinnier appearance and more muscle tone, relaxation, and feeling light and warmth [11,12,13,14,15,16]. In addition, skin diseases, enhanced self-confidence and the role of significant others (such as friends, family and social media) were named especially in studies focusing on adolescents and students [13,15,16,17,18]. Similar to indoor tanning, attractiveness has been the focus of research on intentional outdoor tanning [19,20]. Previous research has also shown that vitamin D synthesis is a further important reason for outdoor tanning, alongside relaxation, feeling of warmth, well-being and mental health [16,19,21].

To address this research gap, nationwide data was used to comprehensively analyze the motivations behind both outdoor and indoor tanning simultaneously. Understanding the reasons for tanning will be instrumental in developing targeted public awareness campaigns. When these campaigns are tailored to the specific motivations for tanning, they will be more effective in educating individuals about the risks associated with UV exposure.

## 2. Materials and Methods

### 2.1. Study Setting

The data for this study were collected within the ninth wave of the National Cancer Aid Monitoring (NCAM-online), which includes a sample of 4156 participants from Germany aged 16 to 65 years [22]. We recruited a quota sample based on sex, age, school education, and federal state via an online panel of the ISO-certified international survey company Bilendi (bilendi.de). The aim of our quota sampling was to align the distribution of key characteristics in our sample with that of the general population in Germany. The online survey took place in November 2024. All participants gave informed consent for participation. The Ethics Committee of the Friedrich-Alexander-Universität Erlangen-Nürnberg (#22-337-S) gave the study ethical approval on 2 November 2022.

### 2.2. Measures

Information collected on the participants’ sociodemographic characteristics included sex (male/female), age, and immigrant background (yes/no based on three questions following Schenk et al. [23]). Further, the participants’ highest level of school education, categorized as low (still at school, no school-leaving qualification or general school qualification), medium (secondary school), and high (high school graduate), as well as the occupational status, categorized as fulltime, parttime, and none, were taken into account. The classification by Fitzpatrick [24] was used to categorize the participants’ self-reported skin type. For this study, the skin types were grouped into skin type categories I to II and III to VI.

The participants’ outdoor tanning behavior was gathered with the question ‘How often do you go out in the sun in order to get tan?’, which could be answered on a 5-point scale ranging from ‘very often’ to ‘never’. For analyses, the answers were grouped into ‘(very) often’, ‘rarely/sometimes’, and ‘never’. Regarding indoor tanning behavior, participants were asked ‘Have you ever used a solarium or a tanning bed?’ with the answer options ‘yes’ and ‘no’. Those who reported experience of indoor tanning were then asked, ‘How often have you used a tanning bed in the last 12 months, counting from today?’, to which they could respond either by ‘not at all’ or by providing a number of uses. These questions allowed us to distinguish between ‘current use’, i.e., use within the last twelve months, ‘former use’, i.e., last use was more than twelve months ago, and ‘never use’. The reasons for outdoor and indoor tanning were each assessed by the same set of motives, with the response options ‘yes’ and ‘no’. The reasons listed were ‘enhancement of attractiveness’, ‘pre-tanning for holidays’, ‘skin diseases’, ‘feeling of light and warmth’, ‘health strengthening’, ‘relaxation’, ‘to get vitamin D’, and ‘physician’s recommendation’. Multiple responses were allowed.

### 2.3. Statistical Analysis

Descriptive statistical methods were initially performed to describe the data collected. In addition, the sample was stratified by skin type. Chi-squared tests were used to detect bivariate associations between the participants’ characteristics and their reasons for each outdoor tanning and indoor tanning. For associations between age and the reasons for tanning, the Mann–Whitney U-test was used. Subsequently, we fitted multivariable logistic regression models for each reason for outdoor and indoor tanning.

In addition, we employed two cluster analyses to identify distinct groups within our dataset based on the similarities in reasons for outdoor and reasons for indoor tanning, respectively. We performed hierarchical cluster analyses (linkage: Ward’s method; distance measure: squared Euclidean distance) on the eight reasons for outdoor tanning and indoor tanning. We used the dendrogram to identify a clear break in the hierarchy and selected the number of clusters accordingly. For outdoor tanning, the dendrogram suggested a five-cluster solution; for indoor tanning, a three-cluster solution. In subsequent analysis, we used a Chi-squared test to identify associations between the clusters and sex as well as tanning behavior. The Mann–Whitney U-test was used to compare age means between the clusters.

For all analyses, an a priori defined *p*-value of *p* < 0.05 was considered significant. All analyses were performed via IBM SPSS Statistics version 29 (IBM Corporation, Armonk, NY, USA).

## 3. Results

Based on the quota sampling, half of the participants were female (Table 1). Mean age was 42.7 (SD: 13.8, Min: 16, Max: 65). Regarding tanning behaviors, 19.2% tanned outdoors (very) often and 63.7% rarely or sometimes.; 13.2% were current users of tanning beds, while 26.1% were former users. Stratified analysis by skin type showed that participants with types I–II had a higher proportion who never tan outdoors than those with types III–VI (24.4% vs. 12.2%); however, 15.5% of participants with types I–II reported tanning (very) often outdoors. The proportion of never users of tanning beds was higher among types I–II than III–VI (63.4% vs. 58.9%), while 12.1% of those with types I–II were current tanning-bed users.

In the subsequent analysis, individuals who did not tan outdoors (17.1%) were excluded from the analysis of outdoor tanning reasons, while those who did not use tanning beds (60.7%) were excluded from the analysis of indoor tanning reasons. For content-related and logical reasons, these subgroups were not queried about their reasons for tanning.

Altogether, 563 individuals did neither tan outdoors nor indoors, while 251 were current indoor tanners and tanned outdoors (very) often (Table 2). Six percent of the participants were current tanning bed users and tanned outdoors (very) often.

**Figure 1:**
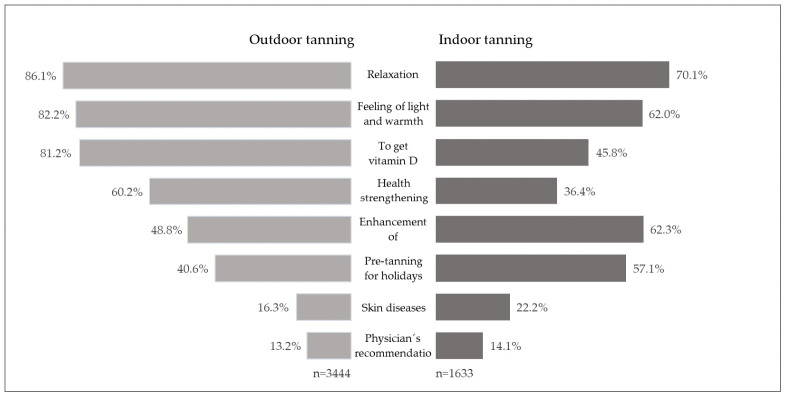
Reasons for outdoor and indoor tanning. Shown are the proportions of those who agreed to the individual reasons.

Reasons for outdoor and indoor tanning differed (Figure 1). While the most frequently named reasons for outdoor tanning were relaxation (86.1%), feeling of light and warmth (82.2%), and to get vitamin D (81.2%), the main reasons for indoor tanning were relaxation (70.1%), enhancement of attractiveness (62.3%), and feeling of light and warmth (62.0%).

Bivariate associations with participants’ characteristics showed similar patterns for outdoor and indoor tanning (Table 3 and Table 4). For outdoor tanning, all reasons, except for increasing vitamin D levels (*p* = 0.103), were more frequently agreed on by participants who tan (very) often compared to those who tan rarely or only sometimes. Similarly, all reasons for using tanning beds were significantly more often agreed upon by current tanning bed users than by former users.

For sex, we found different associations with the motive of increasing vitamin D levels. While more women than men indicated increasing vitamin D levels as a reason for outdoor tanning (85.1% vs. 77.4%, *p* < 0.001, Table 3), fewer women than men selected it as a reason for tanning bed use (42.4% vs. 50.1%, *p* = 0.002, Table 4). Regarding age, we found that those who agreed with the individual reasons were generally younger. However, those who indicated that feeling of light and warmth is a reason for their outdoor tanning were older than those who disagreed (42.8 years vs. 38.9 years, *p* < 0.001).

Immigrant background was positively associated with enhancement of attractiveness and pre-tanning for holidays for both indoor and outdoor tanning. Regarding education, we observed a similar pattern across all reasons with significant differences: agreement was lowest among individuals with low education and highest among those with higher education. An exception for both indoor and outdoor tanning was the reason “physician’s recommendation”: here, the lowest agreement was observed among individuals with medium education. For occupation and skin type, we found mixed results. However, it is worth noting that the reason “physician’s recommendation” had higher agreement among those with fair skin (skin type I–II) than those with darker skin (*p* < 0.001 for indoor and outdoor tanning).

Additional multivariate logistic regression models for each reason for indoor and outdoor tanning support the bivariate findings (Table 5 and Table 6). The strongest associations were found for individual indoor and outdoor tanning, respectively. Regarding outdoor tanning (Table 5), men were more likely than women to indicate skin diseases (OR = 1.267, *p* = 0.020) and a physician’s recommendation (OR = 1.940, *p* < 0.001) as reason, whereas they were less likely than women to indicate enhancement of attractiveness (OR = 0.825, *p* = 0.013), relaxation (0.625, *p* < 0.001), the feeling of light and warmth (OR = 0.788, *p* = 0.015), and vitamin D (OR = 0.612, *p* < 0.001) as reasons for tanning outdoors. Younger age was associated with reporting attractiveness enhancement (OR = 0.979, *p* < 0.001), pre-tanning for holidays (OR = 0.992, *p* = 0.010), skin diseases (OR = 0.970, *p* < 0.001), and physician’s recommendation (OR = 0.963, *p* < 0.001), while the wish for feeling of light and warmth was more common at older ages (OR = 1.267, *p* = 0.020). Participants with an immigrant background were more likely to report tanning outdoors for attractiveness enhancement (OR = 1.485, *p* = 0.002) and pre-tanning for holidays (OR = 1.667, *p* < 0.001), and less likely to experience light and warmth (OR = 0.720, *p* = 0.028). Significant differences could also be identified by education achievement: higher education was positively associated with reporting attractiveness enhancement (OR = 1.604, *p* < 0.001), feeling of light and warmth (OR = 1.931, *p* < 0.001), and relaxation (OR = 1.427, *p* = 0.008) as reasons for outdoor tanning, whereas those with lower education more often indicated skin diseases. Employment was positively associated with pre-tanning for holidays (e.g., fulltime employment: OR = 1.915, *p* < 0.001). Participants with skin types I–II were significantly more likely to report tanning outdoors due to skin disease (OR = 1.628, *p* < 0.001) or on a physician’s recommendation (OR = 1.898, *p* < 0.001), whereas those with types III–VI more often indicated relaxation as the reason.

For indoor tanning, men were more likely than women to cite skin disease (OR = 1.371, *p* = 0.019), perceived health strengthening (OR = 1.741, *p* < 0.001), and a physician’s recommendation (OR = 2.130, *p* < 0.001) as reasons for tanning (Table 6). For older participants pre-tanning for holidays (OR = 1.012, *p* = 0.015) was more relevant as reason for indoor tanning, while attractiveness enhancement (OR = 0.984, *p* = 0.001), skin disease (OR = 0.968, *p* < 0.001), perceived health strengthening (OR = 0.988, *p* = 0.018), and a physician’s recommendation (OR = 0.961, *p* < 0.001) were less relevant. An immigrant background was positively associated with pre-tanning for holidays (OR = 1.669, *p* = 0.008) and negatively associated with indicating a physician’s recommendation (OR = 0.553, *p* = 0.043). Higher education was positively associated with reporting attractiveness enhancement (OR = 1.548, *p* = 0.002) and negatively with reporting relaxation (OR = 0.701, *p* = 0.018) and vitamin D (OR = 0.721, *p* = 0.019) as a reason for indoor tanning. Participants not in employment were more likely than those working full-time to use tanning beds for perceived health strengthening; conversely, employed participants were more likely to report tanning bed use following a physician’s recommendation (e.g., working parttime: OR = 2.391, *p* = 0.014). Skin types I–II were positively associated with indicating skin disease (OR = 1.583, *p* < 0.001) and a physician’s recommendation (OR = 1.794, *p* < 0.001), whereas relaxation-related motives were more frequently reported by those with skin types III–VI.

**Figure 2:**
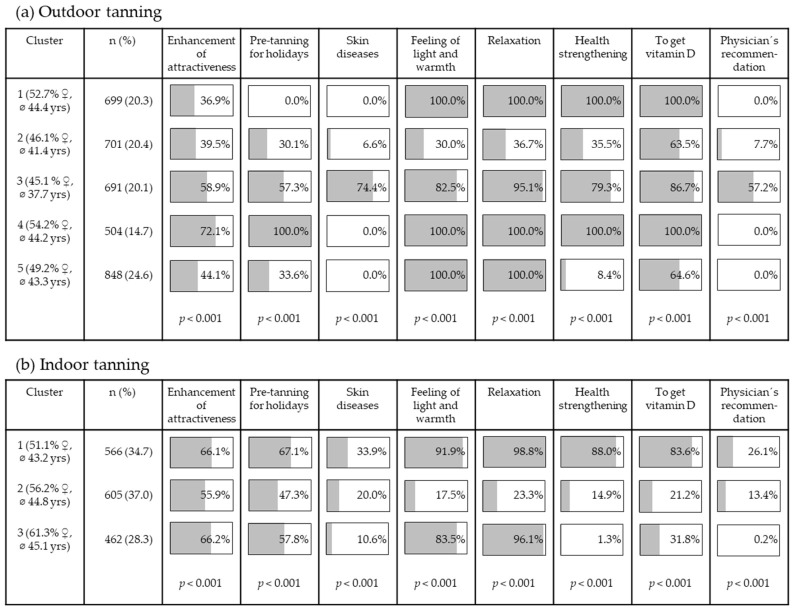
Results of the cluster analyses on reasons for (**a**) outdoor tanning and (**b**) indoor tanning.

For outdoor tanning, cluster analysis revealed five groups of tanners (Figure 2a). The largest cluster (Cluster 5, 24.6%) comprised individuals primarily motivated by wellness-related reasons (i.e., relaxation and the feeling of light and warmth). These wellness-related reasons were also important for individuals in Cluster 1 and Cluster 4. However, those in Cluster 1 also identified increasing vitamin D levels and health enhancement as reasons for outdoor tanning. In addition to these reasons, Cluster 4 indicated pre-tanning as a motivation and showed the highest agreement regarding the enhancement of attractiveness. Compared to Clusters 1 and 5, Cluster 4 included a larger proportion of participants who tan outdoors (very) often (37.0% vs. 16.9% vs. 16.0%). Moreover, Cluster 3 contained a higher proportion of participants who tan outdoors (very) often (36.9%) and had an additional focus on skin diseases, as well as the highest proportion of individuals who reported following a physician’s recommendation.

Regarding indoor tanning, three clusters were identified (Figure 2b). Cluster 1 included the highest proportion of current tanning bed users (49.5%, compared to 23.3% in Cluster 2 and 27.3% in Cluster 3). Cluster 1 exhibited the highest agreement on wellness and health-related items, whereas Cluster 3 predominantly emphasized wellness-related reasons. Cluster 2 comprised a group of individuals who did not show very high agreement with any of the items.

## 4. Discussion

Our study identified that indoor and outdoor tanners differ in their motivations for tanning. While wellness-related reasons (i.e., relaxation and the feeling of light and warmth) were most frequently indicated by both groups, the most frequently named reasons for outdoor tanning included increasing vitamin D levels and health strengthening, while for indoor tanning, the enhancement of attractiveness and pre-tanning were emphasized. However, for both indoor and outdoor tanning reasons, those who tan (very) often outdoors and current tanning bed users showed higher agreement rates than those who tan less frequently or are former tanning bed users. Additionally, our cluster analyses revealed points of connection for potential educational and preventive efforts.

Our results regarding the importance of wellness-related reasons for both indoor and outdoor tanning are consistent with previous studies [11,12,17]. In addition, qualitative studies have explored that well-being is a reason for indoor tanning [25,26,27,28,29,30,31,32]. Further, the increasing importance of getting vitamin D as a reason for outdoor tanning found in a very recent study [21] was confirmed by our results. Contrary to previous research [15,19,20,33], enhancement of attractiveness was not among the most important motivators, as less than half of the participants reported it to be a reason for outdoor tanning. A reason for this different result could be the higher mean age of our sample and the shift away from tanned skin as a beauty ideal. This needs to be investigated in future studies in more detail, since the association between beauty ideals, prevention, and sun protection is complex [34].

For indoor tanning, however, the enhancement of attractiveness was confirmed to be one of the reasons with the highest rate of agreement [11,12,13,14,15,16,33], only surpassed by relaxation. While qualitative studies identified skin disease [25,28,35] and pre-tanning [25,26,32,35,36] as potential motives for indoor tanning, only 22.2% of participants in our sample endorsed skin disease, suggesting it is a less important motivator than previously reported [12], whereas pre-tanning for holidays was more frequently indicated. We cannot determine whether the respondents engaged in pre-tanning for health reasons, specifically to prepare their skin for vacation sun exposure, or to appear more attractive in swimwear. Nonetheless, both possible motivations should be viewed critically. The use of tanning beds is not suitable for pre-tanning, as the composition of the UV rays differs from that of natural sunlight.

Furthermore, getting vitamin D, identified as a reason with increasing importance for indoor tanners, garnered an agreement of 31.1% among German indoor tanners between 2019 and 2022 [11], and attained an agreement of 45.8% in our study. Qualitative studies have also identified vitamin D as a reason for indoor tanning [25,29,30,36]. Interestingly, the bivariate analysis revealed that increasing vitamin D levels as a reason for outdoor tanning was more common among women, while it was more frequently indicated by men as a reason for indoor tanning. For indoor tanning, the association between sex and increasing vitamin D was not significant in multivariate analysis, but it should be investigated further in additional studies to determine whether it persists in other populations and to explore the underlying reasons. We found that men were more likely than women to report skin disease and a physician’s recommendation as reasons for both indoor and outdoor tanning. While a physician’s recommendation may be related to an underlying skin health condition, our data do not allow us to disentangle this relationship.

Regarding the participants’ age and reasons for outdoor tanning, feeling light and warmth was the only reason for which the average age of those who agreed was significantly higher than that of those who did not agree. This aligns with a smaller study in which relaxation was an important reason for outdoor tanning among older individuals [21]. Younger age was associated with reporting attractiveness enhancement, skin disease, and a physician’s recommendation as reasons for both indoor and outdoor tanning. In particular, the link with attractiveness—and potentially with skin conditions such as acne, which are more common in younger people and may be perceived as detracting from appearance—has also been observed in previous studies of indoor tanning among adolescents and young adults [13,16].

The positive association of having an immigrant background with enhancement of attractiveness and pre-tanning for holidays that we found for indoor and outdoor tanning aligns with previous results regarding reasons for indoor tanning [33]. However, unfortunately, we were not able to conduct more in-depth analyses on the immigrant background. In addition, education was positively associated with enhancement of attractiveness (only indoor tanning) and pre-tanning (both indoor and outdoor tanning). However, in a previous study [33], participants with lower education levels were more likely to agree with indoor tanning to enhance one’s attractiveness. Regarding occupation, we found an association with indicating a physician’s recommendation as a reason for both indoor and outdoor tanning. We cannot rule out that some participants interpreted general medical advice to spend more time outdoors (e.g., because they work mainly indoors) as a recommendation to tan. This should be examined in future studies; physicians should not recommend indoor or outdoor tanning given the associated health risks. We also observed that employment status was associated with outdoor pre-tanning. This may reflect that individuals not in employment take holidays less frequently and therefore are less likely to pre-tan for that specific occasion.

Further, fair-skinned participants (skin types I–II) showed higher agreement for physicians’ recommendations and skin diseases as reasons for both indoor and outdoor tanning. This suggests a gap in education about the risks of UV radiation that should be addressed to promote skin cancer prevention. Although individuals with Fitzpatrick skin types I–II reported indoor and outdoor tanning less often than those with types III–VI, they should ideally not tan at all. Following legislation on indoor tanning in Germany, trained staff should actively discourage them from using tanning beds.

The cluster analyses revealed that specific reasons cluster within certain groups for both outdoor and indoor tanning. The observation that the enhancement of attractiveness received agreement across all clusters is an important indication for prevention efforts. It should continue to be emphasized that there is no such thing as a healthy tan [37]. Additionally, the finding that wellness-related reasons are important motivations for both indoor and outdoor tanning across the clusters should be acknowledged. Promoting alternative relaxation methods besides UV exposure seems important (e.g., visiting spas) [21].

Furthermore, the necessity of increasing vitamin D levels through UV radiation emerged as a key reason in some clusters. This was also found to be an important reason for outdoor tanning in another study across the clusters of participants [21] and it has gained importance as a reason for indoor tanning in recent years [11]. Public education on UV exposure and vitamin D supply is warranted [21]. Extensive tanning sessions are not necessary for vitamin D synthesis. For an adult with skin type II, it is generally sufficient in summer to expose the face, hands and arms to the sun for about 12 min, two to three times a week, without covering them or using sunscreen [38]. Additionally, it is not recommended to use tanning beds to achieve an appropriate vitamin D status, as their health risks outweigh the potential benefits [39]. In addition, most tanning beds primarily use UVA radiation for tanning, which does not contribute to vitamin D synthesis but does damage the skin.

Additionally, medical recommendations appear to be a motivator for some groups in seeking a tan. This is concerning, especially since tanning beds should not be used for health-related reasons [40]. Specific UV therapies should be conducted under dermatological supervision, and other light therapies should be considered for individuals with mental health conditions.

### Limitations

Our study comes with several limitations. First, our sample is selective, but quota sampling was used to make the sample as representative as possible regarding sex, age, school education, and federal state for the German population. Second, due to the quantitative nature of our study, potential overlaps between reasons for tanning could not be taken into consideration. For example, a physician may recommend tanning to increase vitamin D levels. In addition, the item on pre-tanning for holidays may have a health (i.e., to prepare the skin for future sunbathing) and an appearance-related aspect (i.e., to get a tan to look better in swimwear). In addition, because we assessed reasons using predefined items, we cannot rule out that some respondents endorsed a reason simply because it was presented, and that it might not have been mentioned in an open-ended format. Moreover, the item on physician’s recommendations may be interpreted broadly; a physician may simply have advised spending more time outdoors, which respondents might have construed as sunbathing. However, despite these possible ambiguities, this study gives a good overview of potential motives for tanning both indoors and outdoors. Third, although we used a question to assess outdoor tanning behavior that has been employed in several previous studies, we acknowledge that the response categories may be subjective and, therefore, be interpreted differently by participants. Our cognitive pretest showed that category-based items to capture outdoor tanning (e.g., never/rarely/sometimes/often/very often) were more comprehensible and easier to answer than specifying numeric tanning frequencies (e.g., times per week/month). Fourth, we cannot rule out recall bias and social desirability, as with growing public awareness about the risks of UV radiation, intentional tanning might be considered irresponsible. The latter bias may have been reduced by the online format of the questionnaire compared to in-person interviewing. Moreover, respondents may overestimate their skin type according to Fitzpatrick’s classification, resulting in a higher prevalence of self-reported types III–VI. Fifth, due to the cross-sectional design of our study, we are not able to draw causal inferences. Sixth, we were unfortunately unable to adjust for sun-protection behaviors, which can modify the effects of UV exposure.

## 5. Conclusions

In view of the rising incidence of melanoma and non-melanoma skin cancer, our data show that the reasons for indoor and outdoor tanning partially differ. However, the overarching similarities provide points of connection for prevention efforts. These include (1) education on the UV exposure sufficient to support adequate vitamin D status, (2) alternatives for relaxation beyond sun exposure and tanning beds, and (3) reconsidering the Western beauty ideal of tanned skin.

(1) Tanning beds are Group 1 carcinogens and therefore neither a safe nor a recommended method for vitamin D synthesis [4]. In addition, intensive tanning in natural sunlight is also not necessary for vitamin D synthesis [38]. Only if a vitamin D deficiency has been diagnosed by a physician is supplementation warranted. This needs to be communicated clearly to the general population.
(2) When there are alternatives to sun exposure and tanning beds (e.g., attending wellness facilities for relaxation or using sunless tanning products to enhance appearance), and tanning is perceived as less relevant, it can contribute to a reduction in both outdoor and indoor tanning, which in turn can help lower the incidence of skin cancer in the long term. Therefore, such potential alternatives to indoor and outdoor tanning should be included in public awareness campaigns.
(3) Given that tanned skin is still seen as attractive and healthy in many Western societies, convincing certain subgroups not to tan remains difficult. A shift in beauty standards is a slow, demanding process that requires time and trusted ambassadors. Hence, we need sustained messaging on the importance of limiting UV exposure.

In summary, comprehensive messaging on sun protection is needed for all ages and settings, beginning in childhood, and should include information on options like skin cancer screening. To prevent skin cancer, it is essential to limit UV exposure and adopt protective strategies.

## Figures and Tables

**Table 1:** Characteristics and tanning behavior of participants.

Variable	n	%	Skin Type				
			I–II		III–VI		*p*-Value
			n	%	n	%	
Sex	4156		1676		2480		<0.001
Female	2074	49.9	727	43.4	1347	54.3	
Male	2074	49.9	944	56.3	1130	45.6	
Diverse *	8	0.2	5	0.3	3	0.1	
Age group (in years)	4156		1676		2480		<0.001
16–25	573	13.8	280	16.7	293	11.8	
26–35	857	20.6	442	26.4	415	16.7	
36–45	851	20.5	346	20.6	505	20.4	
46–55	846	20.4	302	18.0	544	21.9	
56–65	1029	24.8	306	18.3	723	29.2	
Immigrant background	4156		1676		2480		0.053
No	3788	91.1	1545	92.2	2246	90.4	
Yes	368	8.9	131	7.8	237	9.6	
Education	4099		1653		2446		0.282
Low	1056	25.8	425	25.7	631	25.8	
Medium	1349	32.9	523	31.6	826	32.9	
High	1694	41.3	705	42.6	989	40.4	
Occupation	4102		1649		2453		0.189
Fulltime	2356	57.4	919	55.7	1437	58.6	
Parttime	836	20.4	352	21.3	484	19.7	
None	910	22.2	378	22.9	532	21.7	
Outdoor tanning	4156		1676		2480		<0.001
Never	710	17.1	409	24.4	303	12.2	
Rarely/sometimes	2648	63.7	1008	60.1	1640	66.1	
(Very) often	796	19.2	259	15.5	537	21.7	
Indoor tanning	4156		1676		2480		0.014
Never use	2523	60.7	1062	63.4	1461	58.9	
Former use	1083	26.1	411	24.5	672	27.1	
Current use	550	13.2	203	12.1	347	14.0	
Skin type	4156						
I–II	1676	40.3					
III–VI	2480	59.7					

SD = standard deviation; * due to the small case number, diverse individuals were excluded from further analyses.

**Table 2:** Overview of indoor and outdoor tanning behaviors.

		Indoor Tanning			
		Never	Former	Current	
**Outdoor tanning**	Never	13.5% (n = 563)	3.4% (n = 141)	0.2% (n = 8)	17.1% (n = 712)
	Rarely/ sometimes	38.3% (n = 1592)	18.4% (n = 765)	7.0% (n = 291)	63.7% (n = 2648)
	(Very) often	8.9% (n = 368)	4.3% (n = 177)	6.0% (n = 251)	19.2% (n = 796)
		60.7% (n = 2523)	26.1% (n = 1083)	13.2% (n = 550)	100.0% (n = 4156)

**Table 3:** Reasons for outdoor tanning by participants’ characteristics.

	Reasons for Outdoor Tanning																							
	Enhancement of Attractiveness			Pre-Tanning for Holidays			Skin Diseases			Feeling of Light and Warmth			Relaxation			Health Strengthening			To Get Vitamin D			Physician’s Recommendation		
	No %	Yes %	*p*-Value	No %	Yes %	*p*-Value	No %	Yes %	*p*-Value	No %	Yes %	*p*-Value	No %	Yes %	*p*-Value	No %	Yes %	*p*-Value	No %	Yes %	*p*-Value	No %	Yes %	*p*-Value
Sex			0.083			0.548			0.179			0.224			<0.001			0.805			<0.001			<0.001
Female	49.8	50.2		58.9	41.1		84.6	15.4		17.0	83.0		11.8	88.2		39.7	60.3		14.9	85.1		89.5	10.5	
Male	52.7	47.3		59.9	40.1		82.9	17.1		18.5	81.5		15.9	84.1		40.1	59.9		22.6	77.4		84.3	15.7	
Immigrant background			<0.001			<0.001			0.195			0.012			0.422			0.147			0.097			0.015
No	52.5	47.5		60.7	39.3		84.0	16.0		17.2	82.8		13.7	86.3		40.2	59.8		19.2	80.8		87.3	12.7	
Yes	38.6	61.4		47.3	52.7		81.2	18.8		22.9	77.1		15.4	84.6		36.1	64.0		15.4	84.6		82.4	17.6	
Education			<0.001			<0.001			0.887			0.001			0.008			0.499			0.140			0.045
Low	58.7	41.3		64.5	35.5		83.6	16.4		21.2	78.8		16.8	83.2		40.9	59.1		20.6	79.4		88.1	11.9	
Medium	54.8	45.2		60.4	39.6		84.4	15.6		18.7	81.3		14.4	85.6		38.5	61.5		19.5	80.5		88.5	11.5	
High	44.5	55.5		56.5	43.5		84.0	16.1		15.3	84.7		12.1	87.9		40.4	59.6		17.4	82.6		85.4	14.6	
Occupation			<0.001			<0.001			0.055			0.069			0.081			0.509			0.029			<0.001
Fulltime	48.3	51.7		55.3	44.7		82.8	17.2		18.9	81.1		13.4	86.6		39.4	60.6		19.9	80.1		86.0	14.0	
Parttime	51.7	48.4		58.7	41.3		83.2	16.8		15.8	84.2		12.3	87.7		38.6	61.4		15.4	84.6		84.1	15.9	
None	60.7	39.3		72.5	27.5		86.7	13.3		15.9	84.1		16.3	83.7		41.5	58.5		18.6	81.4		91.8	8.2	
Skin type			0.435			0.114			<0.001			<0.001			<0.001			0.758			0.164			<0.001
I–II	52.1	47.9		61.2	38.8		78.6	21.4		22.2	77.8		17.0	83.0		40.2	59.8		17.6	82.4		81.5	18.5	
III–VI	50.7	49.3		58.4	41.6		86.7	13.3		15.2	84.8		12.0	88.0		39.6	60.4		19.5	80.5		89.9	10.1	
Outdoor tanning			<0.001			<0.001			<0.001			0.007			<0.001			<0.001			0.103			<0.001
Rarely/sometimes	57.7	42.3		66.9	33.1		86.5	13.5		18.7	81.3		15.7	84.3		44.0	56.0		19.4	80.6		90.2	9.8	
(Very) often	29.6	70.4		34.7	65.3		74.5	25.5		14.6	85.4		7.7	92.3		26.1	73.9		16.8	83.2		75.6	24.4	

Due to rounding, the values do not always add up to 100%. Significant associations are highlighted in grey.

**Table 4:** Reasons for indoor tanning by participants’ characteristics.

	Reasons for Indoor Tanning																							
	Enhancement of Attractiveness			Pre-Tanning for Holidays			Skin Diseases			Feeling of Light and Warmth			Relaxation			Health Strengthening			To Get Vitamin D			Physician’s Recommendation		
	No %	Yes %	*p*-Value	No %	Yes %	*p*-Value	No %	Yes %	*p*-Value	No %	Yes %	*p*-Value	No %	Yes %	*p*-Value	No %	Yes %	*p*-Value	No %	Yes %	*p*-Value	No %	Yes %	*p*-Value
Sex			0.506			0.877			0.001			0.991			0.278			<0.001			0.002			<0.001
Female	38.4	61.6		42.7	57.3		80.8	19.2		38.0	62.0		31.0	69.0		69.7	30.3		57.6	42.4		90.7	9.3	
Male	36.8	63.2		43.0	57.0		74.1	25.9		38.0	62.0		28.6	71.4		55.9	44.2		49.9	50.1		79.8	20.2	
Immigrant background			0.014			0.003			0.809			0.823			0.584			0.847			0.173			0.321
No	38.6	61.4		44.0	56.0		77.9	22.1		37.9	62.1		29.7	70.3		63.6	36.5		54.7	45.3		85.6	14.4	
Yes	28.7	71.3		31.8	68.2		77.1	22.9		38.9	61.1		31.8	68.2		64.3	35.7		49.0	51.0		88.5	11.5	
Education			<0.001			0.248			0.220			0.551			0.067			0.607			0.145			0.027
Low	45.4	54.6		45.9	54.1		81.2	18.8		35.8	64.2		25.8	74.2		62.9	37.1		50.6	49.4		87.0	13.0	
Medium	37.6	62.4		44.0	56.0		77.8	22.2		39.2	60.8		30.1	69.9		65.8	34.2		57.1	42.9		88.8	11.2	
High	33.2	66.8		40.9	59.1		76.7	23.3		38.6	61.4		32.5	67.5		63.6	36.4		54.5	45.5		83.5	16.5	
Occupation			0.038			0.004			<0.001			0.454			0.701			0.391			0.008			<0.001
Fulltime	35.8	64.2		39.9	60.1		75.8	24.2		39.2	60.8		30.8	69.2		62.3	37.7		50.9	49.1		82.8	17.2	
Parttime	37.5	62.5		44.0	56.0		76.0	24.0		35.7	64.3		28.6	71.4		65.1	34.9		58.9	41.1		85.9	14.1	
None	44.1	55.9		50.8	49.2		86.4	13.6		36.9	63.1		29.2	70.8		66.1	33.9		58.6	41.4		95.9	4.1	
Skin type			0.581			0.984			<0.001			0.013			0.014			0.147			0.130			<0.001
I–II	36.8	63.2		42.8	57.2		72.2	27.9		41.9	58.1		33.6	66.5		61.4	38.6		51.8	48.2		81.1	18.9	
III–VI	38.2	61.8		42.9	57.1		81.3	18.7		35.7	64.3		27.8	72.2		65.0	35.0		55.6	44.4		88.8	11.2	
Indoor tanning			<0.001			<0.001			<0.001			<0.001			<0.001			<0.001			<0.001			<0.001
Former use	42.8	57.2		49.9	50.1		84.6	15.4		43.7	56.3		35.9	64.1		74.6	25.4		64.9	35.1		93.4	6.6	
Current use	27.6	72.4		29.1	70.9		64.5	35.5		26.9	73.1		18.2	81.8		42.0	58.0		33.1	66.9		71.3	28.7	

Due to rounding, the values do not always add up to 100%. Significant associations are highlighted in grey.

**Table 5:** Logistic regression analyses on reasons for outdoor tanning by individual characteristics of participants.

	Reasons for Outdoor Tanning															
	Enhancement of Attractiveness		Pre-Tanning for Holidays		Skin Diseases		Feeling of Light and Warmth		Relaxation		Health Strengthening		To Get Vitamin D		Physician’s Recommendation	
	OR	95–CI	OR	95–CI	OR	95–CI	OR	95–CI	OR	95–CI	OR	95–CI	OR	95–CI	OR	95–CI
Sex																
Female	Ref.		Ref.		Ref.		Ref.		Ref.		Ref.		Ref.		Ref.	
Male	0.83	[0.71; 0.96]	0.85	[0.73; 0.99]	1.27	[1.04; 1.55]	0.79	[0.65; 0.95]	0.63	[0.51; 0.77]	0.94	[0.81; 1.09]	0.61	[0.51; 0.74]	1.94	[1.55; 2.44]
Age	0.98	[0.97; 0.98]	0.99	[0.99; 0.99]	0.97	[0.96; 0.98]	1.03	[1.02; 1.03]	1.000	[0.99; 1.01]	1.01	[1.00; 1.01]	1.00	[0.99; 1.01]	0.96	[0.95; 0.97]
Immigrant background																
No	Ref.		Ref.		Ref.		Ref.		Ref.		Ref.		Ref.		Ref.	
Yes	1.49	[1.15; 1.92]	1.67	[1.30; 2.15]	1.01	[0.73; 1.40]	0.72	[0.54; 0.97]	0.83	[0.59; 1.17]	1.28	[0.99; 1.65]	1.19	[0.86; 1.66]	1.23	[0.87; 1.74]
Education																
Low	Ref.		Ref.		Ref.		Ref.		Ref.		Ref.		Ref.		Ref.	
Medium	1.19	[0.98; 1.45]	1.11	[0.91; 1.36]	0.82	[0.64; 1.07]	1.32	[1.04; 1.67]	1.16	[0.89; 1.50]	1.12	[0.92; 1.35]	1.08	[0.86; 1.37]	0.85	[0.63; 1.16]
High	1.60	[1.32; 1.95]	1.18	[0.97; 1.44]	0.70	[0.54; 0.90]	1.93	[1.52; 2.45]	1.43	[1.10; 1.85]	1.02	[0.84; 1.23]	1.28	[1.02; 1.62]	0.85	[0.64; 1.12]
Occupation																
Fulltime	1.28	[1.05; 1.56]	1.92	[1.55; 2.36]	1.13	[0.86; 1.49]	0.83	[0.64; 1.06]	1.24	[0.96; 1.61]	1.07	[0.88; 1.29]	0.94	[0.74; 1.20]	1.36	[0.97; 1.91]
Parttime	1.11	[0.88; 1.39]	1.62	[1.28; 2.07]	1.16	[0.85; 1.59]	1.05	[0.77; 1.43]	1.25	[0.91; 1.71]	1.10	[0.88; 1.38]	1.10	[0.82; 1.48]	1.86	[1.28; 2.71]
None	Ref.		Ref.		Ref.		Ref.		Ref.		Ref.		Ref.		Ref.	
Skin type																
I–II	0.87	[0.75; 1.01]	0.89	[0.76; 1.04]	1.63	[1.34; 1.98]	0.68	[0.56; 0.81]	0.64	[0.52; 0.79]	1.03	[0.88; 1.29]	1.10	[0.91; 1.32]	1.90	[1.53; 2.36]
III–VI	Ref.		Ref.		Ref.		Ref.		Ref.		Ref.		Ref.		Ref.	
Outdoor tanning																
Rarely/sometimes	Ref.		Ref.		Ref.		Ref.		Ref.		Ref.		Ref.		Ref.	
(Very) often	3.04	[2.54; 3.64]	3.67	[3.08; 4.37]	1.96	[1.60; 2.41]	1.50	[1.19; 1.88]	2.27	[1.70; 3.05]	2.33	[1.94; 2.80]	1.24	[1.00; 1.54]	2.65	[2.12; 3.31]

Statistically significant results are highlighted in grey; CI = confidence interval.

**Table 6:** Logistic regression analyses on reasons for indoor tanning by individual characteristics of participants.

	Reasons for Indoor Tanning															
	Enhancement of Attractiveness		Pre-Tanning for Holidays		Skin Diseases		Feeling of Light and Warmth		Relaxation		Health Strengthening		To Get Vitamin D		Physician’s Recommendation	
	OR	95–CI	OR	95–CI	OR	95–CI	OR	95–CI	OR	95–CI	OR	95–CI	OR	95–CI	OR	95–CI
Sex																
Female	Ref.		Ref.		Ref.		Ref.		Ref.		Ref.		Ref.		Ref.	
Male	0.97	[0.78; 1.22]	0.82	[0.66; 1.02]	1.37	[1.05; 1.79]	0.94	[0.76; 1.18]	1.08	[0.86; 1.37]	1.74	[1.38; 2.20]	1.15	[0.92; 1.44]	2.13	[1.54; 2.95]
Age	0.98	[0.98; 0.99]	1.01	[1.00; 1.02]	0.97	[0.96; 0.98]	1.01	[1.00; 1.02]	0.99	[0.98; 1.00]	0.99	[0.98; 0.99]	0.99	[0.98; 1.00]	0.96	[0.95; 0.97]
Immigrant background																
No	Ref.		Ref.		Ref.		Ref.		Ref.		Ref.		Ref.		Ref.	
Yes	1.44	[0.98; 2.13]	1.67	[1.14; 2.44]	0.75	[0.49; 1.16]	0.89	[0.62; 1.27]	0.72	[0.49; 1.05]	0.76	[0.52; 1.12]	1.01	[0.70; 1.45]	0.55	[0.31; 0.98]
Education																
Low	Ref.		Ref.		Ref.		Ref.		Ref.		Ref.		Ref.		Ref.	
Medium	1.41	[1.07; 1.86]	1.11	[0.85; 1.47]	1.18	[0.83; 1.67]	0.92	[0.69; 1.22]	0.85	[0.63; 1.15]	0.92	[0.69; 1.24]	0.75	[0.57; 0.99]	0.67	[0.43; 1.05]
High	1.55	[1.18; 2.03]	1.20	[0.92; 1.58]	0.99	[0.71; 1.38]	0.92	[0.70; 1.21]	0.70	[0.52; 0.94]	0.87	[0.65; 1.16]	0.72	[0.55; 0.95]	0.77	[0.51; 1.15]
Occupation																
Fulltime	0.95	[0.71; 1.29]	1.33	[0.99; 1.79]	1.17	[0.78; 1.76]	0.83	[0.62; 1.13]	0.77	[0.56; 1.07]	0.65	[0.47; 0.90]	1.01	[0.74; 1.36]	2.30	[1.20; 4.40]
Parttime	1.02	[0.74; 1.42]	1.15	[0.83; 1.58]	1.37	[0.88; 2.13]	1.03	[0.74; 1.43]	0.94	[0.66; 1.33]	0.80	[0.56; 1.13]	0.81	[0.58; 1.13]	2.39	[1.19; 4.79]
None	Ref.		Ref.		Ref.		Ref.		Ref.		Ref.		Ref.		Ref.	
Skin type																
I–II	1.02	[0.82; 1.27]	1.07	[0.86; 1.32]	1.58	[1.23; 2.04]	0.76	[0.62; 0.95]	0.72	[0.57; 0.90]	1.20	[0.96; 1.51]	1.21	[0.97; 1.50]	1.79	[1.31; 2.45]
III–VI	Ref.		Ref.		Ref.		Ref.		Ref.		Ref.		Ref.		Ref.	
Indoor tanning																
Former use	Ref.		Ref.		Ref.		Ref.		Ref.		Ref.		Ref.		Ref.	
Current use	1.67	[1.31; 2.14]	2.66	[2.08; 3.39]	2.19	[1.67; 2.87]	2.39	[1.86; 3.07]	2.48	[1.89; 3.27]	3.65	[2.87; 4.66]	3.34	[2.63; 4.24]	3.55	[2.54; 4.98]

Statistically significant results are highlighted in grey; CI = confidence interval.

## Data Availability

The data presented in this article are not readily available because they are part of an ongoing study. Data will be made available by the authors on reasonable request after study completion.

## Abbreviations

The following abbreviations are used in this manuscript:

SD	Standard deviation
SPSS	Statistical Product and Service Solutions
IBM	International Business Machines Corporation
UV	Ultraviolet
ISO	International Organization for Standardization
NCAM	National Cancer Aid Monitoring
NY	New York
USA	United States of America
